# Editorial: Autonomous Health Monitoring and Assistance Systems With IoT

**DOI:** 10.3389/frobt.2021.611352

**Published:** 2021-03-18

**Authors:** George Azzopardi, Dimka Karastoyanova, Marco Aiello, Christos N. Schizas

**Affiliations:** ^1^ Information Systems Group, Bernoulli Institute for Mathematics, Computer Science and Artificial Intelligence, University of Groningen, Groningen, Netherlands; ^2^ Department of Service Computing, Institute of Architecture of Application Systems, University of Stuttgart, Stuttgart, Germany; ^3^ Department of Computer Science, University of Cyprus, Nicosia, Cyprus

**Keywords:** autonomous health monitoring, elderly, internet of things, early detection and prevention, privacy and scalability

## Introduction

The sustainability of the current healthcare system is being challenged by the growing percentage of the aging population. In addition to the financial sustainability entailed by such a trend, other challenges include the long delays in servicing patients, the consequent late detection of serious health issues, and the necessity of hospitalization. Despite certain risks, the majority of elderly people prefer to age in their own homes. As a matter of fact, studies show that elderly people who choose to keep living independently have longer life expectancies than those who join elderly homes. All these put together emphasize the need to develop technological solutions that autonomously monitor and enhance the well-being of the elderly in their homes.

The uptake of the Internet of Things (IoT) opens new opportunities for how technology can assist people in improving their health and well-being along with improving the cost-effectiveness and quality of health and social services. These technological advancements result in various low-cost sensory equipment that benefit the healthcare of the aging population. Such technological innovation contributes to the development of applications, such as the administration of medication, voice command technologies, telemedicine, and others based on artificial intelligence. In particular, machine learning and predictive analysis combined with IoT will play an important role in the early detection of suspicious signs that, if left untreated, can lead to mobility, mental, and cognitive issues. Other applications may aggregate the high frequency, messy, and intermittent data. Such data can then be incorporated in the Electronic Health Record. To enhance privacy, such records can potentially be based on Blockchain platforms and shared with healthcare professionals. Concerns such as scalability, security, and systems interoperability need to be dealt with urgently in order to achieve sustainable healthcare systems.

The identification of the latest developments in this highly interdisciplinary field of research is the theme of the present Research Topic, that is, autonomous health monitoring and assistive systems with IoT. It aims at covering two main aspects of the topic: information systems and artificial intelligence. The former covers the advancement with respect to IoT-related technologies and information systems in terms of scalability, security, and interoperability, while the latter covers novel approaches and applications based on machine and deep learning.

## Papers Included in This Research Topic

Wang et al. review automatic elderly fall detection systems from the point of view of data collection, data transmission, sensor fusion, data analysis, security, and privacy. They point out that one of the biggest challenges in this field is the collection of a data set with realistic instances of elderlies’ falls. Moreover, it turns out that while various approaches have been proposed, most of them rely on single sensors and work offline. They speculate that better performance is likely to be achieved by focusing on fusing the data from various visual and non-visual portable sensors.

Schiza et al. survey the use that has been made of virtual reality techniques for neurological rehabilitation. Dementia, stroke, spinal cord injury, Parkinson’s, and multiple sclerosis are the diseases considered in the review. Such diseases are the ones that appear most promising for virtual reality applications and have been the most investigated in the literature. The outlook that emerges from the research work of Schiza and colleagues is positive with clear signs of virtual reality being an effective, low-cost, and scalable support for rehabilitation.

Turečková et al. propose a convolutional neural network augmented by deep supervision and attention gates for the segmentation of abdomen computed tomography (CT) images. The system is highly beneficial for medical experts and helps them toward better diagnosis of various pathologies. By means of extensive experiments they conclude that their proposed methodology achieves a reliable organ and tumor segmentation from CT scans. They report state-of-the-art performance on various segmentation tasks. Notable is the increase in precision of tumor segmentation.

Cappiello et al. propose a data model suited for modern health monitoring systems where patient data is generated in the periphery of the network. This is crucial for monitoring patients who are not hospitalized. In the model, the patient-generated data is assessed by quality metrics that are context dependent. The proposed model is assessed using actual user data regarding daily physical activities of healthy young people.

## Conclusion

The articles in this special issue show significant progress toward establishing interdisciplinary research. In particular they encourage interdisciplinary efforts coming from the fields of information systems, distributed systems, artificial intelligence, machine learning, deep learning, software architectures, image processing, virtual reality, monitoring of health, and well-being, among others. The findings presented in these articles also highlight the potential for future advances in each individual field of research as well as at the interface of all relevant research disciplines. If future research places the emphasis on how to serve the healthcare sector and, most importantly, the patients through innovative AI-driven applications, we envision enormous leaps and accumulation of scientific results, software systems, and data. Combined together, they will have a significant societal impact and scientific relevance.

